# Broadening learning communities during COVID-19: developing a curricular framework for telemedicine education in neurology

**DOI:** 10.1186/s12909-021-02979-z

**Published:** 2021-10-29

**Authors:** Christine E. Gummerson, Brian D. Lo, Kori A. Porosnicu Rodriguez, Zoe L. Cosner, Dylan Hardenbergh, Diana M. Bongiorno, Julia Wainger, Katherine Hu, Charlene Gamaldo, Rachel M. E. Salas, Carlos Romo, Doris G. Leung

**Affiliations:** 1 Department of Neurology, Yale University School of Medicine, New Haven, CT USA; 2 Department of Neurology, The Johns Hopkins University School of Medicine, Baltimore, MD USA; 3 Center for Genetic Muscle Disorders, Kennedy Krieger Institute, 707 North Broadway, Room 400A, Baltimore, MD 21205 USA

**Keywords:** Neurology, Medical education, Virtual learning, Telemedicine, Curriculum, Innovation, COVID-19

## Abstract

**Background:**

In response to the cancellation of clinical clerkships due to COVID-19, the Johns Hopkins (JH) Neurology Education Team developed a virtual elective to enhance medical students’ clinical telemedicine skills and foster community between academic institutions.

**Methods:**

This two-week clinical elective, entitled “Virtual Patient Rounds in Neurology,” was administered once in April 2020 and once in May 2020. The curriculum included attending/fellow-led Virtual Rounds, Student Presentations, and Asynchronous Educational Activities. We also developed a new lecture series entitled *JHNeuroChats*, which consisted of live synchronous lectures presented by JH faculty and Virtual Visiting Professors. Trainees and faculty from outside institutions were invited to participate in the *JHNeuroChats*. Students and faculty completed pre- and post-elective surveys to assess the educational impact of the elective. Student’s *t*-tests were used to compare scores between pre- and post-elective surveys.

**Results:**

Seven JH medical students enrolled in each iteration of the elective, and an additional 337 trainees and faculty, representing 14 different countries, registered for the *JHNeuroChats*. We hosted 48 unique *JHNeuroChats*, 32 (66.7%) of which were led by invited Virtual Visiting Professors. At the end of the elective, students reported increased confidence in virtually obtaining a history (*P* < 0.0001) and performing a telehealth neurological physical exam (*P* < 0.0001), compared to the start of the course. In addition, faculty members reported increased confidence in teaching clinical medicine virtually, although these findings were not statistically significant (*P* = 0.15).

**Conclusions:**

Despite the constraints imposed by COVID-19, this virtual Neurology elective increased medical students’ confidence in certain telemedicine skills and successfully broadened our learning community to encompass learners from around the world. As virtual medical education becomes more prevalent, it is important that we are intentional in creating opportunities for shared learning across institutions. We believe that this elective can serve as a model for these future educational collaborations.

**Supplementary Information:**

The online version contains supplementary material available at 10.1186/s12909-021-02979-z.

## Introduction

The 2019 Coronavirus (COVID-19) pandemic has significantly impacted medical education for students across the United States. In March 2020, in accordance with guidelines from the Association of American Medical Colleges, medical schools suspended clinical clerkships and other activities to protect students’ health, maintain physical distancing, and preserve personal protective equipment [1]. As a result, many students found themselves at home without the ability to participate in patient care. Prior to COVID-19, the reported use of online electives in medical education was limited, with only a few curricula published in the medical education literature [2, 3]. However, one meta-analysis found that online undergraduate medical education can be just as effective as in-person education, with noted benefits such as increased flexibility and decreased costs [4]. With this in mind, there was a need to re-evaluate virtual medical education in the context of the pandemic and optimize its implementation [5–8].

In March 2020, the Johns Hopkins (JH) University School of Medicine suspended clinical clerkships and transitioned to a fully online elective curriculum. In response to a call for virtual learning opportunities from educational leaders for Undergraduate Medical Education, the JH Neurology Education Team developed and implemented a comprehensive two-week clinical elective entitled “Virtual Patient Rounds in Neurology.” This elective provided medical students an opportunity to learn about Neurology, gain experience with telemedicine modalities, maintain their clinical skills during the pandemic, and connect with a diverse group of international faculty and peers. We also invited medical students from outside institutions to participate in our synchronous live lecture series (*JHNeuroChats*) as a non-credit learning opportunity.

In this manuscript, we aim to (1) describe the process of developing and implementing the curriculum, (2) present data regarding student and faculty experiences with the curriculum, and (3) highlight the unique ways in which this elective allowed students and faculty from around the world to meaningfully participate in our shared learning community.

## Methods

### Curriculum development team

**Fig. 1:**
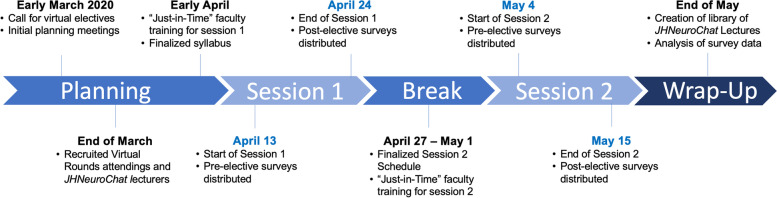
Timeline outlining major events in the development and implementation of the “Virtual Patient Rounds in Neurology” elective from March to May 2020

The “Virtual Patient Rounds in Neurology” elective was developed by the JH Neurology Education Team, composed of the Neurology clerkship directors, the Neurology clerkship coordinator, and a team of JH medical students known as Osler Apprentices (OAs) in Neurology (Additional file 1). OAs are competitively-selected senior medical students at JH who have completed the Neurology clerkship and have a strong interest in medical education. The OA program allows selected students to gain first-hand experience with the pedagogical, curricular, and administrative aspects of pursuing a clinician-educator role at an academic institution [9]. In mid-March 2020, in response to the call for virtual learning opportunities, the JH Neurology Education team started to develop this two-week virtual elective (Fig. 1). Our team met twice a week throughout March to develop the syllabus and determine the associated learning objectives (Table 1).

**Table 1:** Learning objectives for the “Virtual Patient Rounds in Neurology” elective

**Learning Objectives**	
• Review the key signs, symptoms, and examination findings in neurological disorders	
• Practice and refine case presentation and teaching skills	
• Learn the principles of localization, development of a differential diagnosis, and management of neurological diseases	
• Examine implementation strategies for neuroradiological, electrophysiological, and other diagnostic modalities in neurologic disease	
• Engage the medical literature in providing evidence-based management of neurological illnesses	
• Gain experience with certain telemedicine skills in Neurology, including obtaining a focused history and performing the neurological physical exam	

### Course implementation

This two-week elective was offered once in April 2020 and once in May 2020. All aspects of the course were organized by the JH Neurology Education Team without additional financial or administrative resources. Synchronous activities were hosted on Zoom™, a widely used video-conferencing platform with screensharing features. Our Neurology clerkship coordinator created and managed each Zoom™ meeting using institutional student accounts and one departmental account. Our IT department uploaded Zoom™ recordings to a shared website, but was not involved in the day-to-day course activities.

**Fig. 2:**
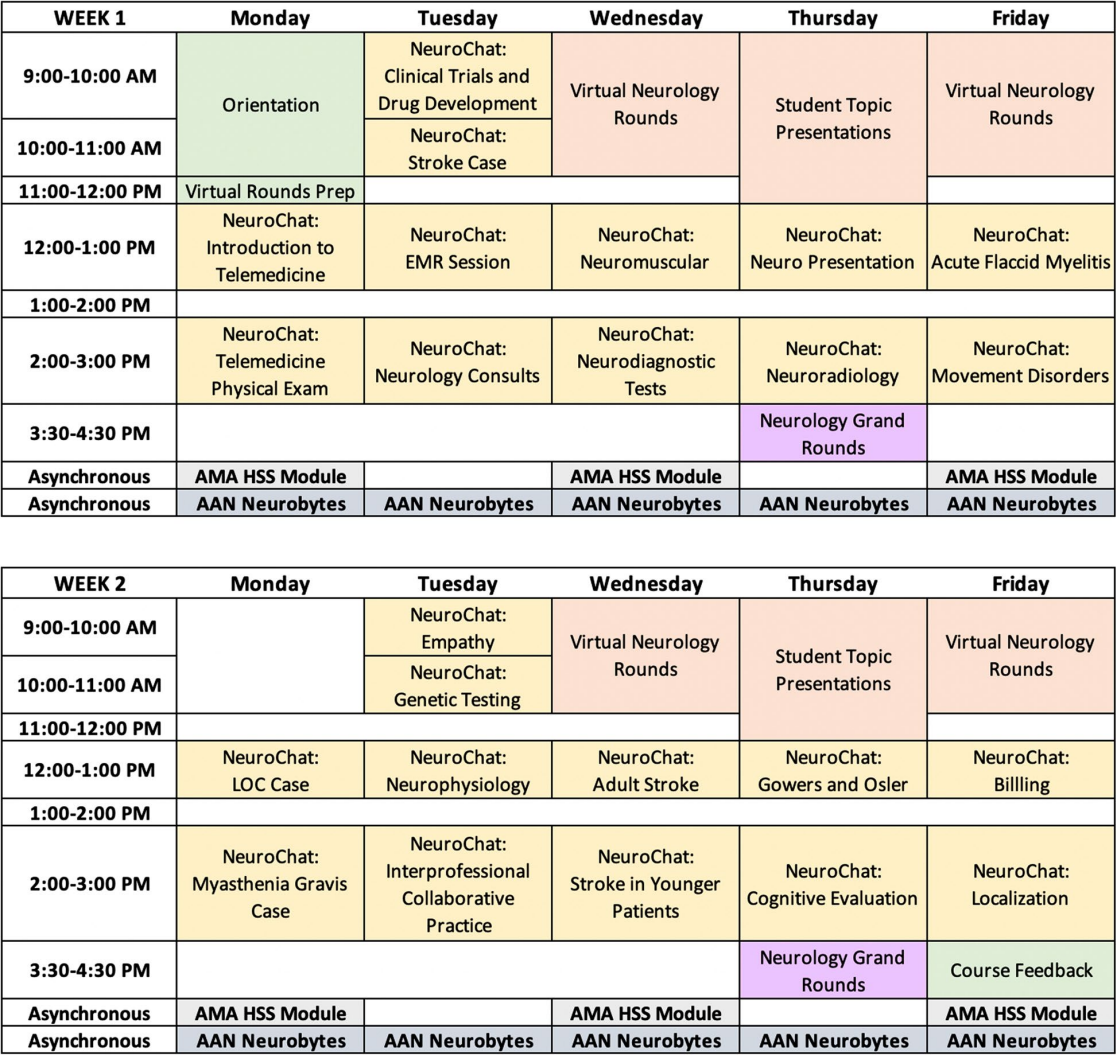
Sample student schedule for the two-week “Virtual Patient Rounds in Neurology” elective

At the start of each elective, a pre-elective survey (Additional file 2) and course schedule (Fig. 2) containing Zoom™ links and passwords were sent to each registered student and faculty member. At the end of each week, JH students met with the course directors to provide feedback, thus allowing for any issues and concerns to be addressed in real time. At the conclusion of the elective, a post-elective survey was distributed to all students and faculty for completion (Additional file 2).

### Curriculum components

The final curriculum for the “Virtual Patient Rounds in Neurology” elective had four components: Virtual Rounds, *JHNeuroChats*, Student Presentations, and a series of Asynchronous Educational Activities (Fig. 2).

#### Virtual Rounds

Virtual Rounds attendings and fellows were recruited from JH based on their interest in medical education and their previous experience working with medical students during the Neurology core clerkship. Prior to each iteration of the elective, a 90-min “Just-in-Time” training session was provided for participating faculty. These sessions were run by the Neurology clerkship directors and focused on familiarizing faculty with the virtual education platform and the concept of Virtual Rounds. Volunteer OAs would briefly present patient cases and one of our team members would ask follow-up questions to simulate the Virtual Rounds experience**.**

Learning groups included a Virtual Rounds attending, two to three JH medical students, and one OA. Patient assignments were distributed by the attending on the first day of the course (Monday). Though each attending conducted rounds slightly differently (Additional file 3), in general, students were responsible for reviewing their patients’ electronic medical records and, where feasible, scheduling and conducting telemedicine visits prior to the Virtual Rounds sessions on Wednesdays and Fridays. Specific information describing the structure of each rounding team is included in Additional file 3. In preparation for rounds, students were invited to schedule one-on-one meetings with OAs to ask questions, practice their oral presentations, and discuss patient notes. During rounds, students presented their patients in the traditional “SOAP” format, with attendings providing complementary teaching on relevant topics. After rounds, students were responsible for submitting a full patient write-up to their attending and OA. Virtual Rounds teams changed each week to allow students to gain exposure to different subspecialties within Neurology.

#### *JHNeuroChats*

The *JHNeuroChats* were designed to be interactive, one-hour-long sessions on a variety of topics within Neurology. *JHNeuroChat* lecturers were recruited from both JH and institutions around the world. Lecturers from outside institutions were appointed “Virtual Visiting Professors.” To ensure that each lecture would be of high quality and targeted towards second- or third-year medical students, we intentionally focused our recruitment efforts on faculty who were career educators or involved with national medical education committees. Care was also taken to ensure diversity with respect to institutional affiliation, sex, gender, race, ethnicity, medical degree (MD or DO), and academic rank. Official invitations were distributed via email approximately 2 weeks in advance of the target lecture date. The course directors and invited lecturers identified *JHNeuroChat* topics ranging from specific topics such as neurological disorders and diagnostic methods, to broader topics such as telemedicine, interprofessional collaborative practice, and health systems science. In addition to JH medical students, trainees (medical students, residents, fellows) and faculty from institutions around the world were invited to attend the *JHNeuroChats* via an online registration form that was distributed widely. JH undergraduate premedical students were also invited to join the *JHNeuroChats* and observe Virtual Rounds [10].

The course coordinator and OAs served as the Zoom™ host and co-host, respectively. Their roles included admitting attendees from the waiting room, monitoring the meeting chat, and aiding with any technological difficulties. The course directors served as moderators of the discussion, introducing each Virtual Visiting Professor and collecting questions from the attendees. Throughout each *JHNeuroChat*, students were able to share questions using the chat function. Course directors and OAs could similarly use the chat box to respond to questions or share additional information pertinent to the ongoing lecture. Mentimeter polls were used during each *JHNeuroChat* to engage students and elicit session-specific feedback [11]. Each session was recorded by the course coordinator and uploaded to a media site to allow for future viewing [12]. At the end of the elective, Virtual Visiting Professors were sent letters of appreciation and guidance on how to reflect the invited visiting professorship on their CVs.

#### Student presentations

Every Thursday, JH medical students delivered 10-15 min presentations on topics informed by their Virtual Rounds patient cases. OAs coordinated topic choices among students and were also available to help students practice their presentations as needed. The presentations were attended by course directors, OAs, and Virtual Rounds faculty.

#### Asynchronous educational activities

JH medical students were provided access to asynchronous educational activities that complemented their synchronous coursework. These assignments included American Medical Association (AMA) Health Systems Science (HSS) modules and American Academy of Neurology (AAN) NeuroByte™ videos. Students were required to submit completion certificates for each module and share take-home points for the NeuroByte™ videos that they reviewed. Independent study time was built into the schedule to facilitate this without conferring undue burden on students.

### Survey design and distribution

To assess the educational impact of the virtual elective, we performed a single-center observational longitudinal cohort analysis of pre- and post-elective surveys that were distributed to all fully enrolled students and faculty. Surveys were developed by the JH Neurology Education Team and were primarily adapted from course evaluations that are regularly used to obtain feedback from students enrolled in the core clerkships at the JH University School of Medicine. The pre- and post-elective surveys were developed solely for the purposes of this study and have not been previously published. Pre-elective surveys were distributed electronically 2 days prior to the start of the course, with results collected on the first day of the course. Post-elective surveys were distributed electronically on the final day of the course, with results collected within 1 week.

Student surveys focused on assessing their goals for the elective and evaluating their level of confidence in virtually obtaining a neurological history and performing a telehealth neurological physical exam. Confidence was assessed on a Likert scale ranging from 1 (not confident) to 5 (very confident), with scores reported as an average ± standard deviation. Student goals were identified through free-text responses, which were independently reviewed by two members of the JH Neurology Education Team and grouped into thematic categories. There were 14 questions in the student pre-survey and 25 questions in the student post-survey (Additional file 2). Surveys were distributed to all 14 JH medical students who were fully enrolled in the elective.

Surveys for Virtual Attendings focused on assessing their comfort with virtual teaching platforms. Similar to the student surveys, confidence for the faculty surveys was assessed on a Likert scale ranging from 1 (not confident) to 5 (very confident), with scores reported as an average ± standard deviation. There were 9 questions in the faculty pre-survey and 8 questions in the faculty post-survey (Additional file 2). Surveys were distributed to all 8 JH faculty members who served as Virtual Rounds attendings.

### Statistical analysis

All surveys were built on Qualtrics and only de-identified data were available to the JH Neurology Education Team. Student’s *t*-tests were used to compare scores between the pre- and post-elective survey. Statistical significance was defined as *P* < 0.05. This study was deemed exempt from convened review by the JH University School of Medicine Institutional Review Board (IRB). Informed consent was waived by the IRB, as only de-identified records obtained from course evaluations were included in the analysis.

## Results

**Fig. 3:**
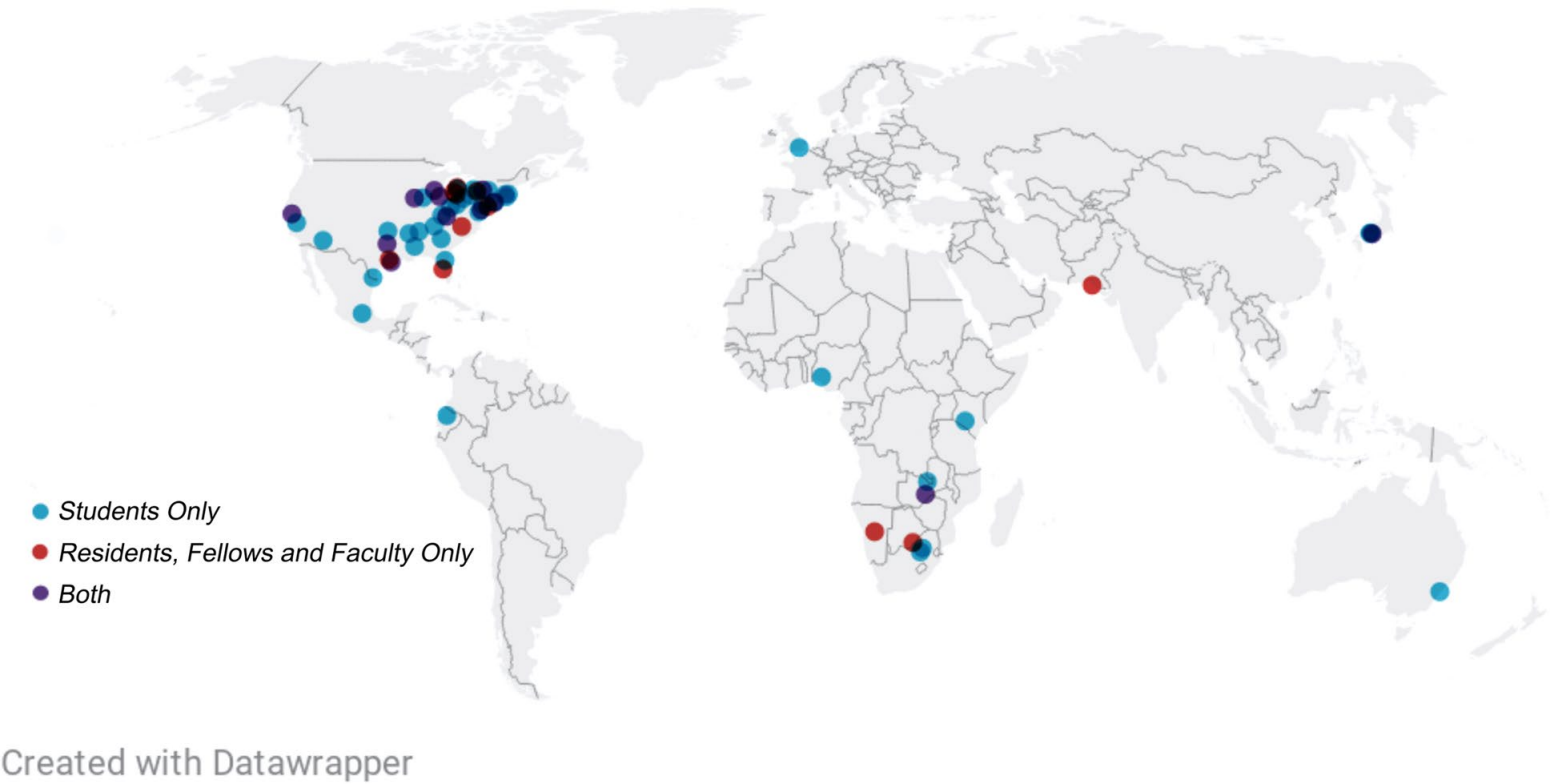
Locations of pre-medical students (*n* = 9) medical students (*n* = 207), residents (*n* = 39), fellows (*n* = 22), and faculty (*n* = 60) from outside institutions who attended the *JHNeuroChats*

In total, 14 medical students from the JH University School of Medicine enrolled in the elective over the two terms that it was offered, and an additional 337 trainees and faculty from institutions around the world signed up to attend one or more of the *JHNeuroChats* (Fig. 3). Among the additional 337 *JHNeuroChat* attendees, 9 (2.7%) were pre-medical students, 207 (61.4%) were medical students, 39 (11.6%) were residents, 22 (6.5%) were fellows, and 60 (17.8%) were faculty. Eight JH Neurology faculty members or fellows led teams as Virtual Rounds attendings for the course. With respect to the *JHNeuroChats*, 48 unique faculty members presented lectures, 32 (66.7%) of whom were Virtual Visiting Professors from outside institutions.

All 14 JH medical students completed the pre- and post-elective surveys. In total, 10 students (71.4%) were MS2s and 4 students (28.6%) were MS3s. Only 3 (21.4%) of these students had previously completed the Neurology core clerkship. JH students reported that their top two goals for the elective were to (1) learn more about Neurology and gain exposure to neurological subspecialties, and (2) maintain their clinical skills during the cancellation of clinical clerkships due to COVID-19. In the post-elective survey, 75% of respondents either slightly agreed or strongly agreed that they achieved their top two stated goals for the rotation [13].

**Fig. 4:**
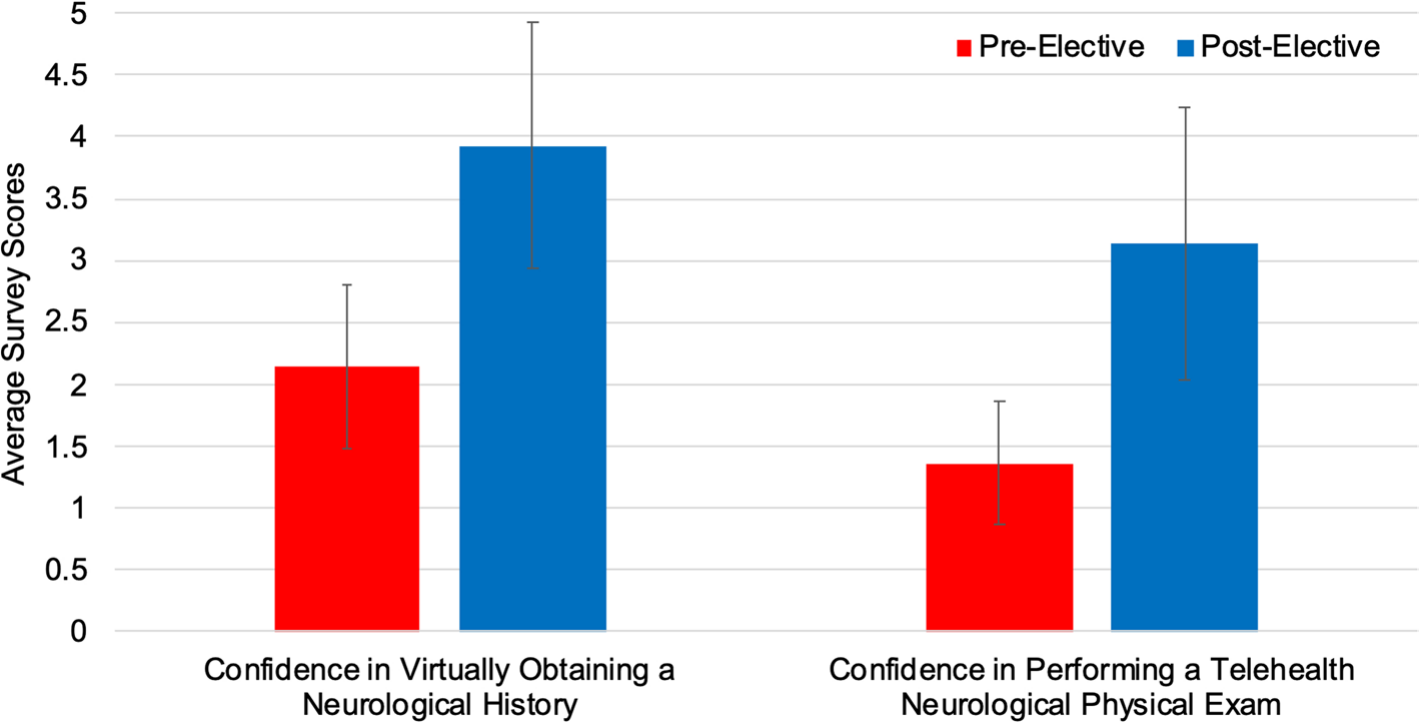
Assessing Johns Hopkins medical student (*n* = 14) confidence in certain telemedicine clinical skills before and after the “Virtual Patient Rounds in Neurology” elective (*P* < 0.0001 for both)

Following completion of the elective, JH medical students reported increased confidence in various telemedicine skills. Students’ scores increased from 2.14 to 3.93 *(P* < 0.0001) for virtually obtaining a complete neurological history, and from 1.36 to 3.14 (*P* < 0.0001) for conducting a telehealth neurological exam (Fig. 4). Further, 57% (*n* = 8) of JH medical students reported that their experience with this elective had either slightly increased or greatly increased their likelihood of choosing a career in Neurology.

At the conclusion of the elective, participating attendings also reported increased confidence in leading Virtual Rounds with students (3.43 to 4.00; *P* = 0.15) and increased comfort with virtual teaching platforms (3.14 to 4.00; *P* = 0.09), although these findings were not statistically significant.

## Discussion

In response to the cancellation of in-person medical student clerkships during the COVID-19 pandemic, the JH Neurology Education Team developed and implemented an innovative virtual clinical elective entitled “Virtual Patient Rounds in Neurology,” the first of its kind at the JH University School of Medicine. While participation in the Virtual Rounds component of the course was restricted to JH medical students due to institutional policy, trainees from institutions around the world were invited to attend the *JHNeuroChats*. As a result, the virtual nature of the elective not only connected JH faculty with JH medical students, but also expanded our learning community beyond institutional borders. After completing the elective, JH medical students reported increased confidence in certain telemedicine skills, including obtaining a complete neurological history and performing a telehealth neurological physical exam. This course also allowed faculty to adapt their clinical education expertise to virtual platforms and gain confidence in navigating virtual teaching modalities.

Conceived of and implemented in a matter of weeks, all components of the course were developed with flexibility and feasibility in mind. The ability to host this course on a widely-available online meeting platform was critical to implementing the course as envisioned. Because all synchronous lectures occurred via Zoom™ meetings, a broader group of students and faculty could participate globally day-to-day and augment their local educational curricula. This format also provided students with the opportunity to learn from a diverse group of experts who could share the nuances of practicing Neurology across institutions and countries. We recorded the *JHNeuroChats* to create a library of resources that faculty and medical students could access as part of their future Neurology core clerkships [12]. By providing access to our library of *JHNeuroChats*, we are able to continue fostering this open learning community and extend our team’s educational outreach beyond the two-week elective.

Though Zoom™ was key to implementing various aspects of the course, the education team also encountered several challenges with the platform. Our team spent a significant amount of time discussing ways to bridge the virtual gap between students and faculty during online meetings. The OAs played an important role in navigating these issues. During Virtual Rounds, OAs served as peer mentors and acted as liaisons between students and faculty. As each OA had already completed multiple core clerkships at JH, they were able to augment students’ educational experiences by providing regular guidance and feedback about rounding expectations within the new virtual medium. Furthermore, during each *JHNeuroChat*, one to two OAs acted as peer facilitators, sharing links to relevant scientific manuscripts or other resources in the chat, while also encouraging participation throughout each discussion. By intentionally incorporating OAs into the elective curriculum, we were able to overcome some of the challenges associated with virtual learning.

Another feature of the course that was particularly important to our team was its curricular focus on telemedicine. The COVID-19 pandemic has already impacted medical education by increasing the utilization of telemedicine in both the inpatient and outpatient clinical settings. Where feasible, telemedicine may soon become a significant modality of patient care, given its lower costs and improved accessibility [14–16]. In light of this, student proficiency in delivering virtual health care will become increasingly important. Very few institutions have dedicated telemedicine curricula for trainees [2, 17], but with the AMA encouraging undergraduate and graduate medical education programs to adopt telemedicine as a core competency, there will be a growing need to train students and faculty in this clinical skill in the future [18, 19]. Given the mutual benefits of incorporating medical students into outpatient clinics, it is essential that students and faculty be well prepared for the transition from in-person to virtual clinical activities [20].

We intentionally organized our course such that the telemedicine *JHNeuroChats* preceded Virtual Rounds, thus allowing students to first learn the principles of telemedicine, and then apply that knowledge to patient interactions under the supervision of an attending physician. For nearly all enrolled JH students, this elective was their first exposure to telemedicine. The increased confidence that students reported in telemedicine will be useful as they return to their core clerkships, many of which will now include telemedicine experiences, and as they prepare for their future careers.

It was also important to provide clinical faculty with opportunities to engage with virtual medical education. Our data demonstrate that training faculty through small-group sessions increased their confidence in leading Virtual Rounds. Though this finding was not statistically significant, likely due to a small sample size, many faculty members commented on how the pre-elective training appropriately prepared them for the opportunities and challenges associated with leading Virtual Rounds. By being intentional in training faculty, we were able to provide them with foundational skills that could be used when teaching students virtually in the future. Many faculty members who were involved in this elective will now be telemedicine preceptors for the JH Neurology core clerkship, and we believe that the training we implemented during this virtual elective will improve future educational experiences for both students and faculty.

Several limitations of this study should be recognized. First, due to our small sample size, we were unable to perform subgroup analyses among JH medical students. This analysis would have been valuable in determining whether students perceived the elective differently based on their prior clinical experiences. Second, we were only able to analyze students’ perception of their improved clinical skills and not their actual abilities. Future studies should seek to determine how this elective influenced students’ ability to obtain a complete history virtually and perform a telehealth neurological physical exam, as assessed by standardized patients and faculty members. Third, it was difficult to track which registrants from outside institutions attended each *JHNeuroChat* session. Fourth, we acknowledge that several factors could impact the generalizability of this curriculum. Though the implementation of this elective did not require funding or significant technological infrastructure, much of the success of our course was predicated upon (1) an institutional commitment to developing and providing credit for online electives, and (2) the availability of senior medical students (OAs) to co-facilitate the course. This curriculum may not be feasible at other institutions that do not have similar support.

In future iterations of the course, we hope to enroll visiting medical students for clinical elective credit. Due to our timeline and institutional policy, it was not possible to achieve this during the initial course. However, we envision a curricular framework wherein institutions could individually host Virtual Rounds and then join together for the synchronous components of the course.

## Conclusions

As a result of the COVID-19 pandemic, virtual clinical education has become more prominent at academic medical institutions around the world. We found that creating a patient-centered virtual Neurology elective increased medical students’ confidence in certain telemedicine skills and enhanced opportunities for shared learning across institutions. As telemedicine becomes further integrated into clinical practice, this curricular framework can serve as a model for future online medical education courses. Additional studies should evaluate the long-term benefits of virtual education and virtual learning communities in clinical medicine.

## Supplementary Information

**Additional file 1.** Overview of the key stakeholders and their associated roles in the “Virtual Patient Rounds in Neurology” elective.

**Additional file 2.** Pre- and post-elective surveys distributed to students and faculty as part of the “Virtual Patient Rounds in Neurology” elective.

**Additional file 3.** Structure of each Virtual Rounds team for the “Virtual Patient Rounds in Neurology” elective.

## Data Availability

The datasets used and/or analyzed during the current study are available from the corresponding author on reasonable request.
