# Supplementary material for: Broadening learning communities during COVID-19: developing a curricular framework for telemedicine education in neurology
Source: BMC Med Educ. 2021 Oct 29;21:549. doi: 10.1186/s12909-021-02979-z (PMC8554502; doi:10.1186/s12909-021-02979-z)
Supplement: Supplementary file 1 — Additional file 1. Overview of the key stakeholders and their associated roles in the “Virtual Patient Rounds in Neurology” elective. [file 12909_2021_2979_MOESM1_ESM.docx]

| **Additional File 1.** Overview of the key stakeholders and their associated roles in the “Virtual Patient Rounds in Neurology” elective | | |
| --- | --- | --- |
| **Title** | **Name** | **Roles** |
| **Course Directors*** | Dr. Rachel M.E. Salas  Dr. Doris G. Leung  Dr. Carlos Romo  Dr. Charlene Gamaldo | - Designed and implemented the elective - Developed the syllabus - Graded assignments for Johns Hopkins students - Developed the pre- and post-elective surveys - Recruited Virtual Rounds Attendings and Virtual Visiting Professors for *JHNeuroChats* - Moderated *JHNeuroChats* and created Mentimeter polls for each session - Led feedback sessions with students and faculty - Sent thank you notes, CV annotations, and feedback to Virtual Visiting Professors and Virtual Rounds Attendings |
| **Osler Apprentices*** | Dr. Christine Gummerson  Brian D. Lo  Kori A. Porosnicu Rodriguez  Zoe L. Cosner  Dylan Hardenbergh  Diana M. Bongiorno  Julia Wainger | - Designed and implemented the elective - Promoted the elective among Johns Hopkins medical students - Developed the pre- and post-elective surveys - Led “Just-in-Time” faculty training, including a demonstration of Virtual Rounds - Scheduled and organized Virtual Rounds - Served as peer mentors for students during Virtual Rounds - Peer facilitators during *JHNeuroChats* - Coordinated student presentation topics - Analyzed pre- and post-elective survey data |
| **Course Coordinator*** | Bernadette Clark | - Enrolled Johns Hopkins students in the elective - Coordinated final grades for Johns Hopkins students - Served as the primary contact to address student questions and concerns throughout the elective - Distributed Zoom links prior to the start of each elective - Zoom host during *JHNeuroChats* |
| **Undergraduate Student**  **(JH PreDoc)** | Katherine Hu | - Attended *JHNeuroChats* and student presentations - Distributed surveys to *JHNeuroChat* students |
| **Virtual Rounds Attendings** | Dr. Carl Stafstrom  Dr. Kelly Mills  Dr. Yujie Wang  Dr. Eric Kossoff  Dr. Barney Stern  Dr. Carlos Romo  Dr. Kemar Green  Dr. Emily Harrington | - Attended “Just-in-Time” faculty training - Led a Virtual Rounds team of 2-3 Johns Hopkins medical students - Identified patients for students to interview and present during rounds - Provided complementary teaching during Virtual Rounds - Provided feedback on patient write-ups |
| **Virtual Visiting Professors** | 48 Faculty Members | - Presented a one-hour interactive lecture to participating students from around the world |

* Denotes members of The Johns Hopkins Neurology Education Team
