# Supplementary material for: Broadening learning communities during COVID-19: developing a curricular framework for telemedicine education in neurology
Source: BMC Med Educ. 2021 Oct 29;21:549. doi: 10.1186/s12909-021-02979-z (PMC8554502; doi:10.1186/s12909-021-02979-z)
Supplement: Supplementary file 2 — Additional file 2. Pre- and post-elective surveys distributed to students and faculty as part of the “Virtual Patient Rounds in Neurology” elective. [file 12909_2021_2979_MOESM2_ESM.docx]

**Additional File 2.** Pre- and post-elective surveys distributed to students and faculty as part of the “Virtual Patient Rounds in Neurology” elective.

**Student Pre-Elective Survey:**

1. Class year
   - MS2
   - MS3
   - MS4
   - Other: _____
2. Have you already completed the Neurology Core Clerkship?
   - Yes, No
3. Which core clerkships have you completed as of now? Please select all that apply.
   - Medicine
   - Surgery
   - Pediatrics
   - Women’s Health
   - Psychiatry
   - Neurology
   - Emergency Medicine
   - ICU
4. Are you interested in pursuing a career in Neurology?
   - Yes
   - No – interested in another specialty (please specify specialty choice if known ____________)
   - Uncertain
5. What are your goals for this 2-week elective experience? Please provide at least 2 below and record these goals elsewhere to look back on throughout and after the course.
   - [Free Response]
6. What is your primary reason for taking this elective?
   - Interest in neurology
   - Need elective credit to graduate
   - Maintaining clinical skills
   - Other: ________
7. Have you ever taken a virtual course? If so, which course?
   - [Free Response]
8. Do you have any specific concerns about taking a virtual clinical elective?
   - [Free Response]
9. How do you typically prefer to complete coursework?
   - Attend educational sessions in person
   - Watch educational materials online
   - Other (text box)
10. Please rate your confidence in your ability to perform a reliable neurologic physical examination **in person:**
    - Not at all confident, Minimally confident, Average confidence, Above average confidence, Very confident
11. Please rate your confidence in your ability to perform a reliable neurologic physical examination **through telemedicine**
    - Not at all confident, Minimally confident, Average confidence, Above average confidence, Very confident, N/A
12. Please rate your confidence in your ability to interview and obtain a complete and reliable neurologic history **through telemedicine**
    - Not at all confident, Minimally confident, Average confidence, Above average confidence, Very confident, N/A
13. Please rate your confidence in your ability to obtain a complete and reliable neurologic history **through searching the electronic medical record**
    - Not at all confident, Minimally confident, Average confidence, Above average confidence, Very confident
14. Please rate your confidence in your ability to deliver a clear, concise, and thorough oral presentation of a patient’s neurologic history and examination
    - Not at all confident, Minimally confident, Average confidence, Above average confidence, Very confident

**Student Post-Elective Survey:**

1. I received clear learning objectives for the elective
   - Strongly Disagree, Disagree, Neutral, Agree, Strongly Agree
2. My performance was assessed against the learning objectives
   - Strongly Disagree, Disagree, Neutral, Agree, Strongly Agree
3. I had an opportunity to follow a variety of different patients
   - Strongly Disagree, Disagree, Neutral, Agree, Strongly Agree
4. Faculty provided effective teaching during the elective
   - Strongly Disagree, Disagree, Neutral, Agree, Strongly Agree
5. Were you observed taking the relevant portions of the patient history?
   - Yes, No
6. Were you observed performing the relevant portions of the physical or mental status exam through telemedicine?
   - Yes, No
7. Rate the quality of your educational experiences in this elective
   - Poor, Fair, Good, Very Good, Excellent
8. Please rate your confidence on your ability to perform a reliable neurologic physical examination **through telemedicine**
   - Not at all confident, Minimally confident, Average confidence, Above average confidence, Very confident
9. Please rate your confidence on your ability to interview and obtain a complete and reliable neurologic history **through telemedicine**
   - Not at all confident, Minimally confident, Average confidence, Above average confidence, Very confident
10. Please rate your confidence on your ability to obtain a complete and reliable neurologic history **through searching the electronic medical record**
    - Not at all confident, Minimally confident, Average confidence, Above average confidence, Very confident
11. Please rate your confidence on your ability to deliver a clear, concise, and thorough oral presentation of a patient’s neurologic history and examination
    - Not at all confident, Minimally confident, Average confidence, Above average confidence, Very confident
12. Please indicate which of the following activities you participated in during this elective. Select all that apply:
    - Interviewed and obtained a patient history through telemedicine
    - Performed a neurological exam through telemedicine
    - Obtained a patient history through searching the electronic medical record
    - Presented a patient during virtual rounds with an attending present
13. Were there any topics that you wish you were able to learn or practice during the elective?
    - Yes (please add comment below), No
    - [Insert Comment – Free Response]
14. How satisfied were you with virtual rounds?
    - Extremely Dissatisfied, Slightly Dissatisfied, Neutral, Slightly Satisfied, Extremely Satisfied
15. How satisfied were you with learning about telemedicine?
    - Extremely Dissatisfied, Slightly Dissatisfied, Neutral, Slightly Satisfied, Extremely Satisfied
16. How satisfied were you with the online Neurobytes video modules?
    - Extremely Dissatisfied, Slightly Dissatisfied, Neutral, Slightly Satisfied, Extremely Satisfied
17. How satisfied were you with the NeuroChats?
    - Extremely Dissatisfied, Slightly Dissatisfied, Neutral, Slightly Satisfied, Extremely Satisfied
18. How satisfied were you with the online AMA Health Systems Science (HSS) Modules?
    - Extremely Dissatisfied, Slightly Dissatisfied, Neutral, Slightly Satisfied, Extremely Satisfied
19. What were your two favorite NeuroChat topics? Why?
    - [Free Response]
20. At the beginning of this elective, we asked you for the top 2 goals that you hoped to achieve during your rotation with us. Do you agree that you have successfully achieved these goals?
    - Strongly Disagree, Disagree, Neutral, Agree, Strongly Agree
21. How did this elective affect your likelihood of choosing a career in the field of Neurology?
    - Decreased greatly, Decreased slightly, No change, Increased slightly, Increased greatly
22. What were the greatest strengths of this elective?
    - [Free Response]
23. What aspects of this virtual elective could be improved in the future?
    - [Free Response]
24. How effective was Zoom at engaging students?
    - Very ineffective, Slightly ineffective, Neutral, Slightly Effective, Very Effective
25. Any final comments about the elective?
    - [Free Response]

**Faculty Pre-Elective Survey:**

1. Have you ever used an online virtual platform (i.e., telemedicine) to see patients in clinic before?
   - Yes, No
2. Have you ever rounded with students using an online virtual platform before?
   - Yes, No
   - If yes, how? [Free Response]
3. Have you taught or observed a student/trainee perform a neurological exam using a virtual platform before?
   - Yes, No
4. If you answered yes to the previous question(s,) please briefly describe the experience
   - [Free response]
5. Please list any concerns that you may have about the virtual nature of the course:
   - [Free Response]
6. What do you anticipate being the biggest challenge(s) about leading virtual rounds with medical students?
   - [Free Text]
7. Have you ever had formal training in telemedicine?
   - Yes, No
   - If yes, please check when (select all that apply)
     - Medical School
     - Residency
     - Fellowship
     - Attending
     - Never received official training
     - Other [Free Response]
8. Please rate your current level of comfort with virtual teaching platforms.
   - Not at all confident, Minimally confident, Average confidence, Above average confidence, Very confident
9. Please rate your confidence in your ability to lead virtual rounds with students
   - Not at all confident, Minimally confident, Average confidence, Above average confidence, Very confident

**Faculty Post-Elective Survey:**

1. How would you compare the level of student engagement on virtual rounds compared to your previous experience rounding with students in the hospital?
   - Significantly Less, Slightly Less, The Same, Slightly More, Significantly More, Not applicable
2. How often did you experience technical difficulties that impacted your ability to interact/engage with and teach students?
   - Very Often, Fairly Often, Sometimes, Rarely, Never
3. If you answered "Very Often” or “Fairly Often” in the previous question, please explain
   - [Free Text}
4. Please rate your current level of comfort with virtual teaching platforms
   - Not at all confident, Minimally confident, Average confidence, Above average confidence, Very confident, N/A
5. Please rate your confidence in your ability to lead virtual rounds with students
   - Not at all confident, Minimally confident, Average confidence, Above average confidence, Very confident
6. Has your level of comfort with virtual teaching platforms changed from before?
   - [Free Text]
7. Do you have any ideas on ways to improve the virtual elective? Are there any online student engagement tools that you would like to recommend for future versions of this course?
   - [Free Text]
8. Would you be interested in being involved in this elective again in the future as a virtual attending?
   - Yes, No
