# Supplementary material for: Broadening learning communities during COVID-19: developing a curricular framework for telemedicine education in neurology
Source: BMC Med Educ. 2021 Oct 29;21:549. doi: 10.1186/s12909-021-02979-z (PMC8554502; doi:10.1186/s12909-021-02979-z)
Supplement: Supplementary file 3 — Additional file 3. Structure of each Virtual Rounds team for the “Virtual Patient Rounds in Neurology” elective. [file 12909_2021_2979_MOESM3_ESM.docx]

**Additional File 3.** Structure of each Virtual Rounds team for the “Virtual Patient Rounds in Neurology” elective. Virtual Rounds sessions were held via Zoom. Students and attendings were instructed to conceal protected health information during presentations and when reviewing any diagnostic tests. Each rounding team consisted of 1-2 attendings, 1 Osler Apprentice, and 2-3 medical students. In developing this chart, descriptions of rounding structures were collected immediately after the elective and analyzed for key words.

| **Components of Virtual Rounds** | | | | | | | | |
| --- | --- | --- | --- | --- | --- | --- | --- | --- |
| **Attending** | **Pre-work** | **Guidance on**  **Clinical Scenario** | **EMR Chart Review** | **Supervised Video or Phone Call** | **Student-**  **Only**  **Video or Phone Call** | **SOAP Format Oral Presentation** | **Attending Teaching Segment** | **Feedback on Written Notes** |
| **#1** | **X** |  | **X** |  | **X** | **X** | **X** | **X** |
| **#2** |  | **X** | **X** |  | **X** | **X** | **X** | **X** |
| **#3** |  | **X** | **X** |  |  | **X** | **X** | **X** |
| **#4** |  | **X** | **X** |  |  | **X** | **X** | **X** |
| **#5** |  | **X** | **X** |  |  | **X** | **X** | **X** |
| **#6** |  | **X** | **X** | **X** | **X** | **X** | **X** | **X** |
| **#7 & 8 (Joint Team)** | **X** | **X** | **X** |  |  | **X** | **X** | **X** |

| **Pre-work** | Included reviewing articles, videos, and downloading phone applications in preparation for rounds. |
| --- | --- |
| **Electronic Medical Record (EMR) Chart Review** | Students were provided with the patient MRN two days before their oral presentation. |
| **Guidance on Clinical Scenario** | Ahead of rounds, attendings would specify the period of time within the EMR that students should focus on and/or which chief concern or overall diagnostic question the student should focus on. |
| **Supervised Video or Phone Call** | Attendings would interview the patient jointly with the student either by video conferencing platform (e.g. Zoom, Skype, FaceTime) or phone, depending on patient and attending preference. |
| **Student-Only Video or Phone Call** | Students would interview the patient either by video conferencing platform (e.g. Zoom, Skype, FaceTime) or phone, depending on patient preference. |
| **SOAP Format Oral Presentation** | Student gave the traditional SOAP (Subjective, Objective, Assessment, Plan) oral presentations. |
| **Attending Teaching Segment** | Attendings would provide teaching following student presentations. This could include highlighting teaching points, reviewing a prepared PowerPoint presentation, and/or reviewing patient diagnostics in greater detail (e.g. neuro-imaging, EEG findings etc.) |
| **Feedback on Written Notes** | Attendings would provide feedback on the two patient notes written by each student. |
